# MTHFR C677T Gene Polymorphism and Head and Neck Cancer Risk: A Meta-Analysis Based on 23 Publications

**DOI:** 10.1155/2015/681313

**Published:** 2015-01-31

**Authors:** Yu-Ming Niu, Mo-Hong Deng, Wen Chen, Xian-Tao Zeng, Jie Luo

**Affiliations:** ^1^Department of Stomatology and Center for Evidence-Based Medicine and Clinical Research, Taihe Hospital, Hubei University of Medicine, Shiyan 442000, China; ^2^The State Key Laboratory Breeding Base of Basic Science of Stomatology & Key Laboratory of Oral Biomedicine, Ministry of Education, Department of Oral and Maxillofacial Surgery, School & Hospital of Stomatology, Wuhan University, Wuhan 430079, China; ^3^Department of Radiology, Taihe Hospital, Hubei University of Medicine, 32 South Renmin Road, Shiyan 442000, China; ^4^Center for Evidence-Based and Translational Medicine, Zhongnan Hospital, Wuhan University, Wuhan 430071, China; ^5^Department of Neurosurgery and Evidence-Based Medicine Center, Taihe Hospital, Hubei University of Medicine, Shiyan 442000, China

## Abstract

*Objective.* Conflicting results on the association between MTHFR polymorphism and head and neck cancer (HNC) risk were reported. We therefore performed a meta-analysis to derive a more precise relationship between MTHFR C677T polymorphism and HNC risk. *Methods.* Three online databases of PubMed, Embase, and CNKI were researched on the associations between MTHFR C677T polymorphism and HNC risk. Twenty-three published case-control studies involving 4,955 cases and 8,805 controls were collected. Odds ratios (ORs) with 95% confidence interval (CI) were used to evaluate the relationship between MTHFR C677T polymorphism and HNC risk. Sensitivity analysis, cumulative analyses, and publication bias were conducted to validate the strength of the results. *Results.* Overall, no significant association between MTHFR C677T polymorphism and HNC risk was found in this meta-analysis (T versus C: OR = 1.04, 95% CI = 0.92–1.18; TT versus CC: OR = 1.15, 95% CI = 0.90–1.46; CT versus CC: OR = 1.00, 95% CI = 0.85–1.17; CT + TT versus CC: OR = 1.01, 95% CI = 0.87–1.18; TT versus CC + CT: OR = 1.11, 95% CI = 0.98–1.26). In the subgroup analysis by HWE, ethnicity, study design, cancer location, and negative significant associations were detected in almost all genetic models, except for few significant risks that were found in thyroid cancer. *Conclusion.* This meta-analysis demonstrates that MTHFR C677T polymorphism may not be a risk factor for the developing of HNC.

## 1. Introduction

Head and neck cancer (HNC) is the sixth most common cancer worldwide. It affects the upper aerodigestive epithelium of the paranasal sinuses, nasal cavity, oral cavity, pharynx, and larynx [1]. In 2008, approximately 633,000 new cases and 355,000 deaths occurred because of HNC particularly in South-Central Asia and Central and Eastern Europe [2, 3]. Treatment options for HNC are complicated and include surgery, radiotherapy, chemotherapy, and biological treatments that decrease the quality of life of patients with functional disabilities and facial abnormalities. HNC is a multifactorial disease that may be caused by various complex factors, including human papilloma virus (HPV) infection, lifestyle, and genetic factors [4].

Smoking and alcohol consumption are the major risk factors of HNC. Genetic mutations may potentially alter the susceptibility of an individual to HNC [5]. However, only a small proportion of vulnerable individuals may develop HNC. To date, genetic mutations such as single nucleotide polymorphisms are important for tumorigenesis and increase the risk of developing HNC and other cancers.

Folate is important in deoxynucleoside synthesis to provide methyl groups and in intracellular methylation reactions [6]. Low folate levels can result in uracil misincorporation during DNA synthesis, leading to chromosomal damage, breaks in DNA strands, impaired DNA repair, and DNA hypomethylation [7]. Methylenetetrahydrofolate reductase (MTHFR) is an important enzyme in folate metabolism. Epidemiological evidence suggests that the genetic variants encoding the enzymes involved in folate metabolism may increase the risk of HNC by altering DNA methylation synthesis and genomic stability. Genetic mutations in MTHFR gene alter folate level and DNA methylation that may lead to hereditary diseases and cancer development [8–10].

MTHFR C677T (Ala222Val) polymorphism may result in cancer development by altering the activity of MTHFR enzyme [11]. In 2002, Weinstein et al. conducted the first study and reported a negative association between MTHFR C677T polymorphism and HNC risk [12]. Since then, numerous studies have been performed to determine the association between MTHFR C677T polymorphism and HNC risk, but the results are conflicting. In 2009, Boccia et al. conducted a meta-analysis of nine published studies [13]. Additional studies on the association between MTHFR C677T polymorphism and HNC risk have been published. Therefore, a comprehensive meta-analysis of all the relevant studies should be performed to predict this association accurately.

## 2. Materials and Methods

### 2.1. Search Strategy and Inclusion Criteria

Three online bibliographic databases (PubMed, Embase, and CNKI) were searched with the following search terms “head and neck cancer,” “oropharyngeal cancer,” “MTHFR,” “methylenetetrahydrofolate reductase,” “polymorphism,” “variant,” and “meta-analysis” in English and Chinese. Relevant studies were manually searched to identify from the references of original studies and review articles on the association between MTHFR C677T polymorphism and HNC risk that were published from 2002 (when the first study on this topic was published) to August 10, 2014. All the selected studies complied with the following three inclusion criteria: (a) case-control study on the MTHFR C677T polymorphism and HNC risk, (b) sufficient published data for estimating the odds ratios (ORs) and 95% confidence intervals (CIs), and (c) only the largest or most recent publication that was selected when multiple studies reported the same or overlapping data [14].

### 2.2. Data Extraction

Two investigators (Niu and Deng) independently extracted the following data from each included study: the first author's name, publication date, country, ethnicity (categorized as Asian, Caucasian, and mixed race), study design, number of cases and controls subjects, Hardy-Weinberg equilibrium (HWE), minor allele frequency (MAF), and cancer location. The information from all included studies was compared in terms of accuracy, and discrepancies were discussed with a third reviewer until consensus was achieved.

### 2.3. Statistical Analysis

Crude ORs with 95% CIs were calculated to assess the strength of the correlation between MTHFR C677T polymorphism and HNC risk. Pooled ORs were calculated for allele contrast model (T versus C), codominant model (TT versus CC, CT versus CC), dominant model (TT + CT versus CC), and recessive model (TT versus CC + CT), respectively. Subgroup analysis was performed to statistically analyze HWE, ethnicity, study design, and cancer location. Heterogeneity assumption was calculated based on the *I* ^2^ statistics with low, moderate, and high *I* ^2^ values of 25%, 50%, and 75%, respectively [15, 16]. OR estimation of each models was calculated by using the fixed-effects model (Mantel-Haenszel method) if the *I* ^2^ ≤ 50% (which indicated a lack of heterogeneity) [17]. Otherwise, a random-effects model (the DerSimonian and Laird method) was used [18]. Potential publication bias was estimated by the Egger's linear regression test [19]. Statistical analyses were performed using STATA version 11.0 (Stata Corporation, College Station, TX, USA). Two-sided *P* values were used, and *P* < 0.05 was considered statistically significant.

## 3. Results

### 3.1. Study Characteristics

**Figure 1:**
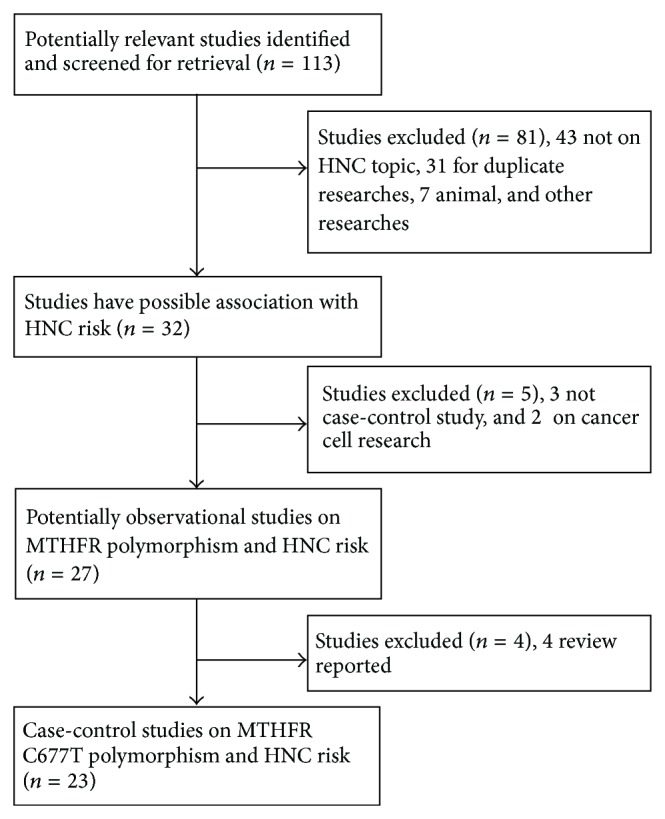
Flowchart of the study selection process.

One hundred thirteen articles were retrieved by literature search. After a careful evaluation, twenty-three related case-control studies on the relationship between MTHFR C677T polymorphism and HNC risk were included in this meta-analysis (Figure 1) [12, 20–41].  Table 1 presents the main characteristics of these studies. Of the 23 studies, 9 studies focused on Asian populations [20, 24, 28, 29, 31, 33, 37–39], 10 studies described Caucasian populations [21–23, 25, 27, 30, 32, 35, 40, 41], and 4 studies assessed mixed populations [12, 26, 34, 36]. The diverse genotyping methods included PCR-RFLP and TaqMan, and the genotypic distribution of the controls was consistent with the HWE in all except four studies [23, 25, 26, 39].

### 3.2. Meta-Analysis

**Figure 2:**
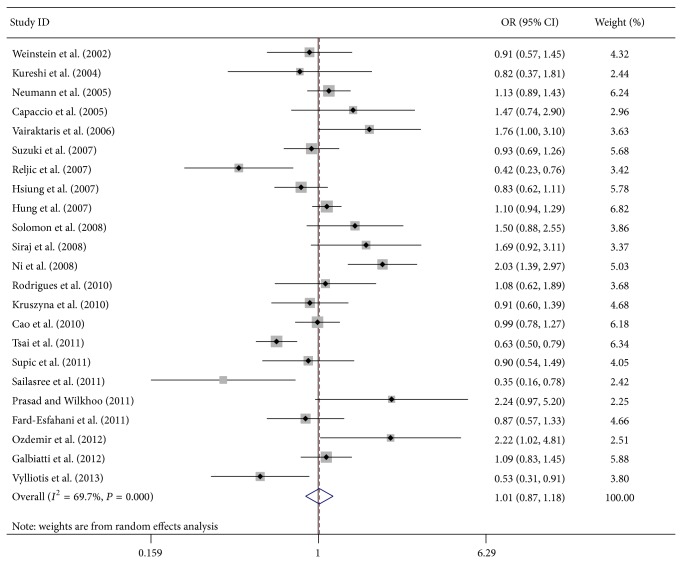
OR of head and neck cancer associated with MTHFR C677T polymorphism for the CT + TT versus CC model in total.

The main results of this meta-analysis and heterogeneity test are presented in Table 2. Overall, no significant association between MTHFR C677T polymorphism and HNC risk was found in this meta-analysis (T versus C: OR = 1.04, 95% CI = 0.92–1.18, *P* = 0.55, *I* ^2^ = 72.5%; TT versus CC: OR = 1.15, 95% CI = 0.90–1.46, *P* = 0.26, *I* ^2^ = 56.8%; CT versus CC: OR = 1.00, 95% CI = 0.85–1.17, *P* = 0.99, *I* ^2^ = 65.8%; CT + TT versus CC: OR = 1.01, 95% CI = 0.87–1.18, *P* = 0.86, *I* ^2^ = 69.7% (Figure 2); TT versus CC + CT: OR = 1.11, 95% CI = 0.98–1.26, *P* = 0.10, *I* ^2^ = 49.8%). Subsequent analysis of the HWE studies showed similar lack of associations between MTHFR C677T polymorphism and HNC risk (T versus C: OR = 1.05, 95% CI = 0.92–1.21, *P* = 0.47, *I* ^2^ = 75.0%; TT versus CC: OR = 1.14, 95% CI = 0.88–1.48, *P* = 0.34, *I* ^2^ = 61.4%; CT versus CC: OR = 1.02, 95% CI = 0.88–1.19, *P* = 0.76, *I* ^2^ = 60.3%; CT + TT versus CC: OR = 1.03, 95% CI = 0.88–1.20, *P* = 0.69, *I* ^2^ = 68.4%; TT versus CC + CT: OR = 1.11, 95% CI = 0.89–1.39, *P* = 0.35, *I* ^2^ = 52.2%). Further, stratified analysis of ethnicity, study design, cancer location, and smoking habits showed no significant association between MTHFR C677T polymorphism and HNC risk. Notable, slight increased risks were found in the risk of developing thyroid cancer (T versus C: OR = 1.30, 95% CI = 1.03–1.65, *P* = 0.04, *I* ^2^ = 43.9%; TT versus CC: OR = 2.06, 95% CI = 1.04–4.10, *P* = 0.04, *I* ^2^ = 0.0%).

### 3.3. Sensitivity Analysis and Cumulative Analysis

**Figure 3:**
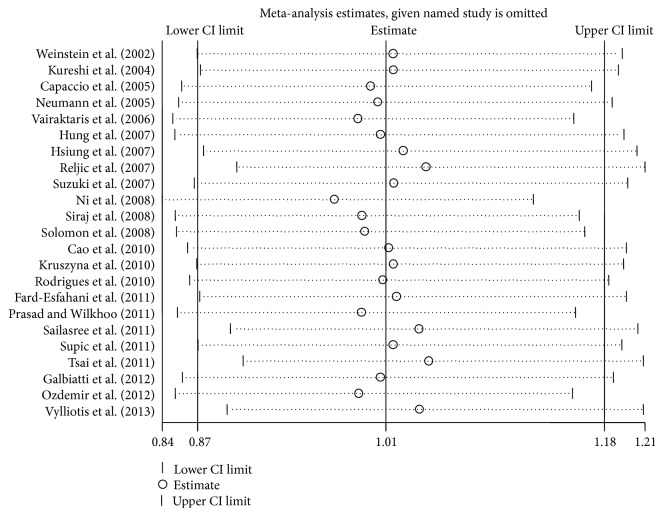
Sensitivity analysis through deletion of one study at a time to reflect the influence of the individual dataset to the pooled ORs in CT + TT versus CC model.

**Figure 4:**
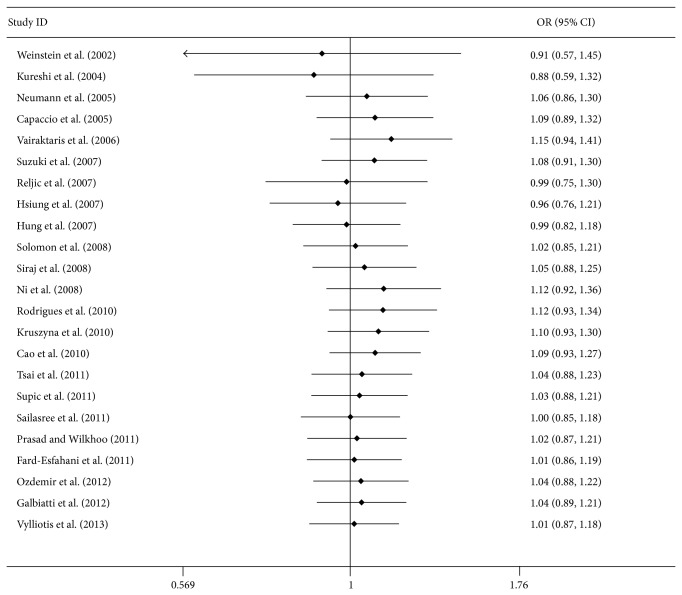
Cumulative meta-analyses according to publication year in CT + TT versus CC model.

Each study included in this meta-analysis was deleted one by one to determine the effect of an individual dataset to the pooled ORs; the results were consistent in all of the research genetic models (Figure 3 for the dominant model), indicating that our results are statistically robust (Table 3 for the dominant model). In the cumulative meta-analysis, the results always showed negative association with the increasing number of studies (Figure 4 for the dominant model).

### 3.4. Publication Bias

**Figure 5:**
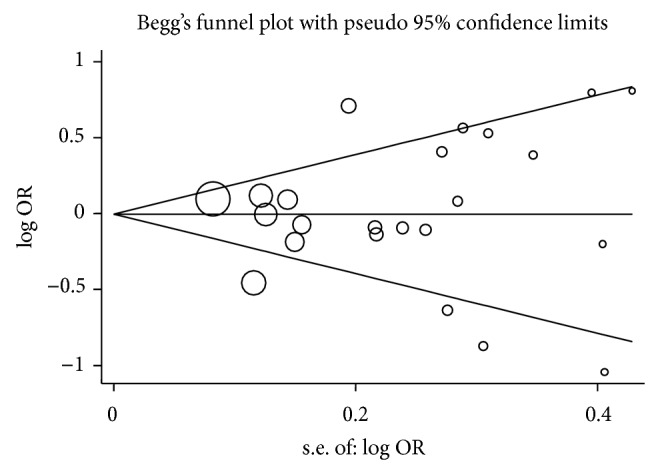
Funnel plot analysis to detect publication bias for CT + TT versus CC model. Each point represents a separate study for the indicated association.

Funnel plot and Egger's test were performed to estimate the publication bias among the included studies. Shapes of the funnel plots for all genetic models did not reveal any asymmetrical evidence (Figure 5 showed the funnel plots for the dominant model in all populations). The result was further supported by the data with Egger's test. No significant publication bias was found in this meta-analysis (*P* = 0.85 for T versus C; *P* = 0.86 for TT versus CC; *P* = 0.91 for CT versus CC; *P* = 0.72 for CT + TT versus CC; *P* = 0.97 for TT versus CC + CT).

## 4. Discussion

MTHFR irreversibly catalyzes the conversion of 5,10-methylenetetrahydrofolate to 5-methyltetrahydrofolate, which is a cosubstrate in the transmethylation of homocysteine to methionine. Methionine is the precursor of S-adenosyl-L-methionine, which is the primary methyl donor during DNA methylation process [42, 43]. In another metabolic reaction, 5,10-methylenetetrahydrofolate is involved in the conversion of deoxyuridylate monophosphate to deoxythymidylate monophosphate. Low levels of 5,10-methylenetetrahydrofolate would result in increasing the amounts of uracil incorporated in DNA to replace thymine, thereby increasing the ratio of point mutations and resulting in DNA breakage [6]. All of these factors are important in cancer development.

Molecular studies have shown that genetic susceptibility is one of the most important risk factors for cancer development. MTHFR gene is mapped in chromosome 1p36.3, is composed of 11 exons and 10 introns, and encodes a 77 KD protein [44, 45]. MTHFR gene C677T polymorphism, which is characterized by the transition of cytosine to thymine, leads to an amino acid change from alanine (Ala) to valine (Val) at codon 222 in exon 4. Previous studies have shown individuals with mutant homozygous 677TT genotype and heterozygous 677CT genotype showed approximately 30% and 65% activities of the MTHFR enzyme, respectively, compared with individuals with wild-type 677CC genotype [11]. Both heterozygous (CT) and homozygous (TT) variants possibly increase enzyme thermolability, reduce MTHFR enzyme activity, and decrease folate concentrations in plasma and red blood cells [46].

To date, large numbers of studies have investigated the association between the MTHFR C677T polymorphism and cancer risks, but the results are inconsistent. The MTHFR C677T variant is a possible risk factor of pancreatic [46], esophageal [47], and breast cancers [48] but exerts a possible protective effect against colorectal cancer [49]. However, the MTHFR C677T variant is not associated with lung [50] and prostate cancers [51].

In 2002, Weinstein et al. observe no association between MTHFR C677T polymorphism and HNC (oral cancer) risk in a Puerto Rican population. Since then, many studies have assessed this association but have obtained inconsistent results. Solomon et al. [28] found that the mutation in homozygous 677TT genotype is associated with a high risk of oral squamous cell carcinoma among Indian heavy drinkers (OR = 3.0; 95% CI = 2.02–4.0). Ni et al. [29] also found that the individuals with 677CT and 677TT genotype had a 1.66-fold (95% CI: 1.08–2.52) and 3.35-fold (95% CI: 2.07–5.54) increased risk of developing laryngeal squamous cell carcinoma, respectively, compared with those who had 677CC genotype in a Chinese population. Vairaktaris et al. [23] supposed that mutations in* MTHFR* slightly increased the risk of oral cancers. Capaccio et al. [22] and Neumann et al. [21] observed the same results for oropharyngeal cancer and HNC among individuals with the CT genotype. In contrast, some studies indicated that the T allele exerts a protective effect against HNC. Sailasree et al. [33] demonstrated that the 677 (CT + TT) genotype was associated with a significant 3-fold reduction in the risk of oral cancer (95% CI = 0.16–0.78) in Indian patients. Tsai et al. [31] showed that the MTHFR 677CT and 677TT genotypes exerted protective effects against oral cancer in Taiwan patients (95% CI = 0.54–0.81 and 95% CI = 0.41–0.86, resp.). Moreover, Reljic et al. [25] also reported a decreased risk tendency for 677CT genotype in a Croatian population. However, other studies have shown no significant association between MTHFR C677T polymorphism and HNC risk [12, 20, 24, 26, 27, 30, 32, 34]. In the stratified analysis with drinking and smoking status, the T allele is also considered as an increased risk factor [24, 32].

This meta-analysis included 23 related studies involving 4,955 cases and 8,805 controls. No significant association was found in all of the genetic models and stratified analysis based on the HWE, ethnicity and study design, and cancer location, expect for few significant risks that were found in thyroid cancer. These results are consistent with two previous meta-analysis on MTHFR gene polymorphism and HNC and oral cancer risk by Boccia et al. [13] in 2009 and Zhuo et al. [52] in 2012, respectively. These meta-analyses included only 9 and 6 studies, respectively. Because of the small sample size and inadequate stratified analysis, further review and meta-analysis with larger sample sizes are necessary to accurately predict the associations between MTHFR C677T polymorphism and HNC risk.

There were some limitations in this meta-analysis. First, these results are based on unadjusted estimates that lack original data from the included studies. Therefore, the evaluation of the gene-environment interactions during HNC development was limited. Second, MTHFR C677T polymorphism was not analyzed in combination with other related genes involved in folate metabolism, such as methionine synthase (MTR), methionine synthase reductase (MTRR), and adjacent polymorphic locus (A1298C), and the effect of gene-gene interactions of MTHFR C677T polymorphism on HNC development was not illustrated clearly. Third, information of folate intake was not obtained, and the influence of folate on the association between MTHFR C677T polymorphism and HNC risk was not explained. Fourth, very few studies included in this meta-analysis involve the smoking and drinking status of patients, and the interaction between gene mutation and effect of environmental factors was not be evaluated accurately. Fifth, heterogeneity existed in all of the genetic models in the total population in our meta-analysis. And the subgroup analyses were conducted to decrease or prevent the occurrence of heterogeneity. The random-effects model was used to estimate the combined effect size when significant heterogeneity was observed.

Despite these limitations, no publication bias was observed. Sensitivity analysis also indicated that the included studies provided consistent and robust results.

## 5. Conclusion

In summary, no significant association was found between the MTHFR C677T polymorphism and HNC risk. Therefore, large-scale case-control and population-based studies involving potential gene-gene and gene-environment interactions are necessary to investigate the association further.

## Figures and Tables

**Table 1:** Characteristics of case-control studies included in the meta-analysis.

							Genotype distribution									
First author	Year	Country/region	Racial descent	Source of controls	Case	Control	Case			Control			Genotyping type	*P* for HWE	MAF	Location
							CC	CT	TT	CC	CT	TT				
Weinstein [12]	2002	Puerto Rico	Mixed	Population control	135	146	67	53	15	69	62	15	PCR-RFLP	0.85	0.32	Oral
Kureshi [20]	2004	Pakistan	Asian	NA	50	54	32	18	0	32	18	4	PCR-RFLP	0.52	0.24	HN
Neumann [21]	2005	USA	Caucasian	Hospital control	537	545	258	244	35	278	216	51	PCR-RFLP	0.34	0.29	HN
Capaccio [22]	2005	Italy	Caucasian	Population control	65	100	18	33	14	36	46	18	PCR-RFLP	0.62	0.41	HN
Vairaktaris [23]	2006	Greece	Caucasian	Population control	110	120	28	76	6	45	65	10	PCR-RFLP	0.04	0.35	Oral
Suzuki [24]	2007	Japan	Asian	Hospital control	237	711	88	113	36	252	331	128	TaqMan	0.29	0.41	HN
Reljic [25]	2007	Croatia	Caucasian	Population control	81	102	45	27	9	35	59	8	PCR-RFLP	0.01	0.37	HN
Hsiung [26]	2007	USA	Mixed	Population control	277	524	128	149		218	306		NA	NA	NA	HN
Hung [27]	2007	Europe	Caucasian	Hospital control	779	2530	373	327	79	1272	1025	233	TaqMan	0.20	0.30	HN
Solomon [28]	2008	India	Asian	NA	126	100	48	55	23	48	42	10	PCR-RFLP	0.86	0.31	Oral
Siraj [35]	2008	Saudi Arabia	Caucasian	Population control	49	511	30	18	1	372	126	13	PCR-RFLP	0.55	0.15	Thyroid
Ni [29]	2008	China	Asian	Hospital control	207	400	48	95	64	152	187	61	PCR-RFLP	0.78	0.39	Laryngeal
Rodrigues [36]	2010	Brazil	Mixed	Population control	100	100	44	43	13	46	40	14	PCR-RFLP	0.28	0.34	HN
Kruszyna [30]	2010	Poland	Caucasian	Population control	131	250	69	52	10	126	104	20	PCR-RFLP	0.82	0.29	Laryngeal
Cao [37]	2010	China	Asian	Population control	511	552	310	169	32	334	188	30	PCR-RFLP	0.60	0.23	NP
Tsai [31]	2011	Taiwan	Asian	Hospital control	620	620	391	186	43	322	236	62	PCR-RFLP	0.06	0.29	Oral
Supic [32]	2011	Serbia	Caucasian	Hospital control	96	162	50	32	14	80	66	16	PCR-RFLP	0.66	0.30	Oral
Sailasree [33]	2011	India	Asian	Hospital control	101	138	92	8	1	108	29	1	PCR-RFLP	0.53	0.11	Oral
Prasad [38]	2011	India	Asian	NA	97	241	86	10	1	228	12	1	PCR-RFLP	0.07	0.03	Thyroid
Fard-Esfahani [39]	2011	Iran	Asian	Hospital control	154	198	69	71	14	82	108	8	multiplex PCR	<0.01	0.31	Thyroid
Ozdemir [40]	2012	Turkey	Caucasian	NA	60	50	28	25	7	33	14	3	Real-time PCR	0.38	0.20	Thyroid
Galbiatti [34]	2012	Brazil	Mixed	Population control	322	531	130	147	45	226	250	55	PCR-RFLP	0.24	0.34	HN
Vylliotis [41]	2013	Greek/Germany	Caucasian	Population control	110	120	76	28	6	65	45	10	PCR-RFLP	0.58	0.27	Oral

HWE in controls.

MAF: minor allele frequency in controls.

NA: not applicable.

HN: head and neck.

NP: nasopharyngeal.

**Table 2:** Summary ORs and 95% CI of MTHFR C677T polymorphism and head and neck cancer risk.

	T versus C				TT versus CC				CT versus CC				CT + TT versus CC				TT versus CC + CT			
	OR	95% CI	*P*	*I* ^2^ (%)^a^	OR	95% CI	*P*	*I* ^2^ (%)^a^	OR	95% CI	*P*	*I* ^2^ (%)^a^	OR	95% CI	*P*	*I* ^2^ (%)^a^	OR	95% CI	*P*	*I* ^2^ (%)
Total	1.04	0.92–1.18	0.55	72.5	1.15	0.90–1.46	0.26	56.8	1.00	0.85–1.17	0.99	65.8	1.01	0.87–1.18	0.86	69.7	1.11	0.98–1.26	0.10	49.8
HWE	1.05	0.92–1.21	0.47	75.0	1.14	0.88–1.48	0.34	61.4	1.02	0.88–1.19	0.76	60.3	1.03	0.88–1.20	0.69	68.4	1.11	0.89–1.39	0.35	52.2
Ethnicity																				
Asian	1.03	0.78–1.36	0.83	86.0	1.29	0.73–2.28	0.38	79.6	0.96	0.73–1.25	0.75	70.8	1.00	0.74–1.36	0.99	80.4	1.28	0.80–2.06	0.30	74.0
Caucasian	1.39	0.89–1.20	0.65	49.1	1.04	0.85–1.27	0.66	0.0	1.04	0.81–1.35	0.75	69.5	1.05	0.83–1.33	0.67	66.3	1.01	0.84–1.22	0.90	0.0
Other	1.08	0.92–1.27	0.36	0.0	1.25	0.87–1.78	0.23	0.0	1.00	0.79–1.27	0.98	0.0	0.96	0.81–1.15	0.68	0.0	1.24	0.89–1.74	0.21	0.0
Design																				
PB	1.01	0.90–1.14	0.84	29.5	1.16	0.92–1.46	0.21	0.0	0.95	0.76–1.20	0.68	60.1	0.96	0.80–1.15	0.64	53.5	1.18	0.95–1.74	0.14	0.0
HB	0.98	0.77–1.25	0.86	87.9	1.09	0.69–1.74	0.71	82.9	0.94	0.72–1.22	0.72	79.1	0.96	0.72–1.27	0.76	84.3	1.07	0.72–1.58	0.74	79.2
Location																				
Oral	0.88	0.67–1.17	0.38	75.3	0.85	0.64–1.12	0.25	48.0	0.81	0.57–1.15	0.24	70.7	0.84	0.59–1.20	0.33	74.4	0.91	0.70–1.20	0.52	34.0
Laryngeal	1.33	0.68–2.60	0.40	90.6	1.82	0.51–6.44	0.353	86.1	1.22	0.70–2.13	0.48	70.5	1.37	0.63–3.00	0.43	86.8	1.64	0.64–4.17	0.30	77.9
Thyroid	1.30	1.03–1.65	0.04	43.9	2.06	1.04–4.10	0.04	0.0	1.48	0.84–2.62	0.17	65.9	1.53	0.92–2.53	0.10	60.5	2.02	1.04–3.92	0.04	0.0

^a^Test for heterogeneity.

PB: population based; HB: hospital based.

**Table 3:** Sensitivity analysis through deleting each study to reflect the influence of the individual dataset to the pooled ORs in CT + TT versus CC model.

Study omitted	Estimate	95% conf. interval	
Weinstein et al. (2002) [12]	1.0189941	0.87112659	1.1919612
Kureshi et al. (2004) [20]	1.0193014	0.87365657	1.1892264
Capaccio et al. (2005) [22]	1.0022544	0.85940462	1.1688484
Neumann et al. (2005) [21]	1.007629	0.85724384	1.184396
Vairaktaris et al. (2006) [23]	0.99266464	0.85276967	1.155509
Hung et al. (2007) [27]	1.0095826	0.85412776	1.193331
Hsiung et al. (2007) [26]	1.0266064	0.87583935	1.2033266
Reljic et al. (2007) [25]	1.0438555	0.90103817	1.2093099
Suzuki et al. (2007) [24]	1.0195761	0.86892009	1.1963534
Ni et al. (2008) [29]	0.97470093	0.84459507	1.1248491
Siraj et al. (2008) [35]	0.9954946	0.85466665	1.1595275
Solomon et al. (2008) [28]	0.99778956	0.85548556	1.1637648
Cao et al. (2010) [37]	1.0159856	0.86369467	1.1951293
Kruszyna et al. (2010) [30]	1.0192744	0.87083119	1.1930214
Rodrigues et al. (2010) [36]	1.0113139	0.86534274	1.1819084
Fard-Esfahani et al. (2011) [39]	1.0215796	0.87302911	1.1954068
Prasad and Wilkhoo (2011) [38]	0.99519187	0.85606372	1.1569312
Sailasree et al. (2011) [33]	1.0387301	0.89613956	1.2040093
Supic et al. (2011) [32]	1.0191994	0.87169868	1.1916586
Tsai et al. (2011) [31]	1.0461444	0.90586507	1.2081468
Galbiatti et al. (2012) [34]	1.0096141	0.85997182	1.1852955
Ozdemir et al. (2012) [40]	0.99327004	0.85463184	1.1543981
Vylliotis et al. (2013) [41]	1.0391399	0.89383554	1.2080653
Combined	1.0136127	0.87153344	1.178854

## References

[1] Parkin D. M., Bray F., Ferlay J., Pisani P. (2005). Global cancer statistics, 2002. *CA Cancer Journal for Clinicians*.

[2] Ferlay J., Shin H. R., Bray F., Forman D., Mathers C., Parkin D. M. (2008). Estimates of worldwide burden of cancer in 2008: GLOBOCAN 2008. *International Journal of Cancer*.

[3] Jemal A., Bray F., Center M. M., Ferlay J., Ward E., Forman D. (2011). Global cancer statistics. *CA: A Cancer Journal for Clinicians*.

[4] Ragin C. C. R., Modugno F., Gollin S. M. (2007). The epidemiology and risk factors of head and neck cancer: a focus on human papillomavirus. *Journal of Dental Research*.

[5] Argiris A., Karamouzis M. V., Raben D., Ferris R. L. (2008). Head and neck cancer. *The Lancet*.

[6] Blount B. C., Mack M. M., Wehr C. M. (1997). Folate deficiency causes uracil misincorporation into human DNA and chromosome breakage: implications for cancer and neuronal damage. *Proceedings of the National Academy of Sciences of the United States of America*.

[7] Duthie S. J. (1999). Folic acid deficiency and cancer: mechanisms of DNA instability. *British Medical Bulletin*.

[8] Han Y., Pan Y., Du Y. (2011). Methylenetetrahydrofolate reductase C677T and A1298C polymorphisms and nonsyndromic orofacial clefts susceptibility in a southern Chinese population. *DNA and Cell Biology*.

[9] Liu A. Y., Scherer D., Poole E. (2013). Gene-diet-interactions in folate-mediated one-carbon metabolism modify colon cancer risk. *Molecular Nutrition and Food Research*.

[10] Mayne S. T., Risch H. A., Dubrow R. (2001). Nutrient intake and risk of subtypes of esophageal and gastric cancer. *Cancer Epidemiology Biomarkers and Prevention*.

[11] Frosst P., Blom H. J., Milos R. (1995). A candidate genetic risk factor for vascular disease: a common mutation in methylenetetrahydrofolate reductase. *Nature Genetics*.

[12] Weinstein S. J., Gridley G., Harty L. C. (2002). Folate intake, serum homocysteine and methylenetetrahydrofolate reductase (MTHFR) C677T genotype are not associated with oral cancer risk in Puerto Rico. *Journal of Nutrition*.

[13] Boccia S., Boffetta P., Brennan P. (2009). Meta-analyses of the methylenetetrahydrofolate reductase C677T and A1298C polymorphisms and risk of head and neck and lung cancer. *Cancer Letters*.

[14] Little J., Bradley L., Bray M. S. (2002). Reporting, appraising, and integrating data on genotype prevalence and gene-disease associations. *American Journal of Epidemiology*.

[15] Lau J., Ioannidis J. P. A., Schmid C. H. (1997). Quantitative synthesis in systematic reviews. *Annals of Internal Medicine*.

[16] Higgins J. P. T., Thompson S. G. (2002). Quantifying heterogeneity in a meta-analysis. *Statistics in Medicine*.

[17] Mantel N., Haenszel W. (1959). Statistical aspects of the analysis of data from retrospective studies of disease. *Journal of the National Cancer Institute*.

[18] DerSimonian R., Laird N. (1986). Meta-analysis in clinical trials. *Controlled Clinical Trials*.

[19] Egger M., Smith G. D., Schneider M., Minder C. (1997). Bias in meta-analysis detected by a simple, graphical test. *British Medical Journal*.

[20] Kureshi N., Ghaffar S., Siddiqui S., Salahuddin I., Frossard P. M. (2004). Head and neck cancer susceptibility: a genetic marker in the methylenetetrahydrofolate reductase gene. *Journal for Oto-Rhino-Laryngology and Its Related Specialties*.

[21] Neumann A. S., Lyons H. J., Shen H. (2005). Methylenetetrahydrofolate reductase polymorphisms and risk of squamous cell carcinoma of the head and neck: a case-control analysis. *International Journal of Cancer*.

[22] Capaccio P., Ottaviani F., Cuccarini V., Cenzuales S., Cesana B. M., Pignataro L. (2005). Association between methylenetetrahydrofolate reductase polymorphisms, alcohol intake and oropharyngolaryngeal carcinoma in northern Italy. *Journal of Laryngology and Otology*.

[23] Vairaktaris E., Yapijakis C., Kessler P. (2006). Methylenetetrahydrofolate reductase polymorphism and minor increase of risk for oral cancer. *Journal of Cancer Research and Clinical Oncology*.

[24] Suzuki T., Matsuo K., Hasegawa Y. (2007). One-carbon metabolism-related gene polymorphisms and risk of head and neck squamous cell carcinoma: case-control study. *Cancer Science*.

[25] Reljic A., Simundic A.-M., Topic E., Nikolac N., Justinic D., Stefanovic M. (2007). The methylenetetrahydrofolate reductase (MTHFR) C677T polymorphism and cancer risk: the Croatian case-control study. *Clinical Biochemistry*.

[26] Hsiung D. T., Marsit C. J., Houseman E. A. (2007). Global DNA methylation level in whole blood as a biomarker in head and neck squamous cell carcinoma. *Cancer Epidemiology Biomarkers and Prevention*.

[27] Hung R. J., Hashibe M., McKay J. (2007). Folate-related genes and the risk of tobacco-related cancers in Central Europe. *Carcinogenesis*.

[28] Solomon P. R., Selvam G. S., Shanmugam G. (2008). Polymorphism in ADH and MTHFR genes in oral squamous cell carcinoma of Indians. *Oral Diseases*.

[29] Ni X., Tai J., Ma L.-J. (2008). Association between genetic polymorphisms in methylenetetrahydrofolate reductase and risk of laryngeal squamous cell carcinoma. *Chinese Journal of Otorhinolaryngology Head and Neck Surgery*.

[30] Kruszyna Ł., Lianeri M., Rydzanicz M., Gajęcka M., Szyfter K., Jagodziński P. P. (2010). Polymorphic variants of folate metabolism genes and the risk of laryngeal cancer. *Molecular Biology Reports*.

[31] Tsai C.-W., Hsu C.-F., Tsai M.-H. (2011). Methylenetetrahydrofolate reductase (MTHFR) genotype, smoking habit, metastasis and oral cancer in Taiwan. *Anticancer Research*.

[32] Supic G., Jovic N., Kozomara R., Zeljic K., Magic Z. (2011). Interaction between the MTHFR C677T polymorphism and alcohol-impact on oral cancer risk and multiple DNA methylation of tumor-related genes. *Journal of Dental Research*.

[33] Sailasree R., Nalinakumari K. R., Sebastian P., Kannan S. (2011). Influence of methylenetetrahydrofolate reductase polymorphisms in oral cancer patients. *Journal of Oral Pathology & Medicine*.

[34] Galbiatti A. L. S., Ruiz M. T., Rodrigues J. O. (2012). Polymorphisms and haplotypes in methylenetetrahydrofolate reductase gene and head and neck squamous cell carcinoma risk. *Molecular Biology Reports*.

[35] Siraj A. K., Ibrahim M., Al-Rasheed M. (2008). Polymorphisms of selected xenobiotic genes contribute to the development of papillary thyroid cancer susceptibility in Middle Eastern population. *BMC Medical Genetics*.

[36] Rodrigues J. O., Galbiatti A. L. S., Ruiz M. T. (2010). Polymorphism of methylenetetrahydrofolate reductase (MTHFR) gene and risk of head and neck squamous cell carcinoma. *Brazilian Journal of Otorhinolaryngology*.

[37] Cao Y., Miao X.-P., Huang M.-Y. (2010). Polymorphisms of methylenetetrahydrofolate reductase are associated with a high risk of nasopharyngeal carcinoma in a smoking population from Southern China. *Molecular Carcinogenesis*.

[38] Prasad V. V. T. S., Wilkhoo H. (2011). Association of the functional polymorphism C677T in the methylenetetrahydrofolate reductase gene with colorectal, thyroid, breast, ovarian, and cervical cancers. *Onkologie*.

[39] Fard-Esfahani P., Fard-Esfahani A., Saidi P., Fayaz S., Mohabati R., Majdi M. (2011). An increased risk of differentiated thyroid carcinoma in Iran with the 677C→T homozygous polymorphism in the MTHFR Gene. *Cancer Epidemiology*.

[40] Ozdemir S., Silan F., Hasbek Z. (2012). Increased T-allele frequency of 677 C>T polymorphism in the methylenetetrahydrofolate reductase gene in differentiated thyroid carcinoma. *Genetic Testing and Molecular Biomarkers*.

[41] Vylliotis A., Yapijakis C., Nkenke E. (2013). Effect of thrombosis-related gene polymorphisms upon oral cancer: a regression analysis. *Anticancer Research*.

[42] Stern L. L., Mason J. B., Selhub J., Choi S.-W. (2000). Genomic DNA hypomethylation, a characteristic of most cancers, is present in peripheral leukocytes of individuals who are homozygous for the C677T polymorphism in the methylenetetrahydrofolate reductase gene. *Cancer Epidemiology Biomarkers and Prevention*.

[43] Kawakami K., Ruszkiewicz A., Bennett G., Moore J., Watanabe G., Iacopetta B. (2003). The folate pool in colorectal cancers is associated with DNA hypermethylation and with a polymorphism in methylenetetrahydrofolate reductase. *Clinical Cancer Research*.

[44] Goyette P., Pai A., Milos R. (1998). Gene structure of human and mouse methylenetetrahydrofolate reductase (MTHFR). *Mammalian Genome*.

[45] Rozen R. (1997). Genetic predisposition to hyperhomocysteinemia: deficiency of methylenetetrahydrofolate reductase (MTHFR). *Thrombosis and Haemostasis*.

[46] Mazaki T., Masuda H., Takayama T. (2011). Polymorphisms and pancreatic cancer risk: a meta-analysis. *European Journal of Cancer Prevention*.

[47] Langevin S. M., Lin D., Matsuo K. (2009). Review and pooled analysis of studies on MTHFR C677T polymorphism and esophageal cancer. *Toxicology Letters*.

[48] Zhang J., Qiu L.-X., Wang Z.-H. (2010). MTHFR C677T polymorphism associated with breast cancer susceptibility: a meta-analysis involving 15,260 cases and 20,411 controls. *Breast Cancer Research and Treatment*.

[49] Kennedy D. A., Stern S. J., Matok I. (2012). Folate intake, *MTHFR* polymorphisms, and the risk of colorectal cancer: a systematic review and meta-analysis. *Journal of Cancer Epidemiology*.

[50] Zhang Y., Qiang Chen G., Ji Y. (2012). Quantitative assessment of the effect of MTHFR polymorphisms on the risk of lung carcinoma. *Molecular Biology Reports*.

[51] Stevens V. L., Rodriguez C., Sun J., Talbot J. T., Thun M. J., Calle E. E. (2008). No association of single nucleotide polymorphisms in one-carbon metabolism genes with prostate cancer risk. *Cancer Epidemiology Biomarkers and Prevention*.

[52] Zhuo X., Ling J., Zhou Y., Zhao H., Song Y., Tan Y. (2012). Polymorphisms of MTHFR C677T and A1298C association with oral carcinoma risk: a meta-analysis. *Cancer Investigation*.

